# microRNA-503 inhibits gastric cancer cell growth and epithelial-to-mesenchymal transition

**DOI:** 10.3892/ol.2014.1868

**Published:** 2014-02-11

**Authors:** YANG PENG, YAN-MIN LIU, LU-CHUN LI, LU-LU WANG, XIAO-LING WU

**Affiliations:** Department of Gastroenterology, Second Affiliated Hospital of Chongqing Medical University, Chongqing 400010, P.R. China

**Keywords:** microRNA-503, epithelial-to-mesenchymal transition, growth, gastric cancer

## Abstract

Epithelial-to-mesenchymal transition (EMT) is believed to be associated with cancer cell malignancy, and also to cause cancer invasion and metastasis. Recent evidence indicates that small non-protein coding RNA [microRNAs (miRNAs/miRs)] may act as powerful regulators of EMT. The present study aimed to systematically delineate miR-503 expression in gastric cancer and analyse the function of miR-503 in gastric cancer EMT. In the present study, miR-503 expression was detected in gastric cancer cell lines and gastric cancer tissues by quantitative polymerase chain reaction. Gastric cancer cell migration, invasion and proliferation capabilities were analysed by Transwell, MTT and clonability assays. The expression of mesenchymal markers, including fibronectin, vimentin, N-cadherin, SNAIL and the epithelial marker, E-cadherin, was examined by immunoblot analysis following miR-503 transfection. miR-503 expression was found to be reduced in gastric cancer cell lines compared with normal gastric mucosa cell lines, and the expression of miR-503 was upregulated in non-metastatic-derived gastric cancer cell lines compared with metastatic-derived lines. miR-503 expression levels were significantly reduced in tumour tissues in comparison with adjacent normal mucosa tissues, and the miR-503 expression levels in patients with metastases were significantly lower than those in patients without. miR-503 inhibited gastric cancer cell migration, invasion and proliferation. Fibronectin, vimentin, N-cadherin and SNAIL protein levels were decreased, but E-cadherin expression was increased in an AGS cell line transfected with miR-503. Taken together, the present findings indicate that miR-503 acts as a novel tumour suppressor gene in gastric cancer and can inhibit EMT in gastric cancer cells.

## Introduction

microRNAs (miRNAs/miRs) are non-coding RNA molecules, 21–23 nucleotides long, that regulate gene expression at the post-transcriptional level (1–3). miRNA expression profiling analyses have revealed a global downregulation of mature miRNA levels in primary human tumours relative to normal tissues (4,5). Thus, miRNAs may function as tumour suppressors or oncogenes, and dysregulated miRNA expression may contribute to tumour cell metastasis.

Metastasis is a complex, multi-step and dynamic biological event, and a critical process in the metastatic cascade is the epithelial-to-mesenchymal transition (EMT). EMT is regulated by a variety of signalling pathways that originate from the stroma that surrounds cancer cells; these pathways are activated by cytokines, including transforming growth factor-β (TGFβ), hepatocyte growth factor, platelet-derived growth factor, epidermal growth factor and integrin engagement, all of which converge at the level of key transcription factors, ZEB, SNAIL and TWIST (6). Recently, the mechanisms behind EMT have been elucidated, and the pathways involved in epithelial marker downregulation and the corresponding mesenchymal marker upregulation have been better characterised (7). One area of significant progress has been the identification of the critical roles of miRNAs in these processes.

The concept of miRNAs as powerful regulators of EMT has transformed the conventional narrative of carcinoma progression, and several miRNAs have now been described as crucial EMT regulators. These miRNAs dynamically affect the balance between EMT and the reverse process, termed mesenchymal-to-epithelial transition (MET) (8). The overexpression of miR-7 has been shown to partially reverse EMT to MET in gastric cancer by targeting the insulin-like growth factor-1 receptor (IGF1R) (9). TGFβ appears to play a dominant role by directly activating the ZEB, SNAIL and TWIST transcription factors; the master regulators of EMT (6). A number of studies profiling miRNA expression have been conducted to identify candidate miRNAs with possible roles in TGFβ-induced EMT (10–14). Several miRNAs have been shown to directly target families of EMT transcription factors, including the miR-200 family; the members of which target ZEB1 and ZEB2 (15). Certain signalling pathways are also involved in EMT. Du *et al* identified that miR-34a downregulation promoted EMT by targeting the Notch signalling pathway in tubular epithelial cells (16).

Recently, Xu *et al* identified that the miR-503 expression level was downregulated in endometrial cancer (EEC) cells, and that a relatively high miR-503 level in EEC tissues indicates a longer EEC patient survival time (17). In addition, our previous study revealed that miR-503 expression was downregulated in gastric carcinoma using gene chip analysis (18). Using the Miranda prediction tool, (http://www.microrna.org), it was predicted that miR-503 may be upstream of EMT regulator proteins, including Notch and IGF1R (19). The results indicate that miR-503 may be an oncomiR and a regulator of EMT. Therefore, further systemic delineation of miR-503 expression and its function in gastric cancer is required.

## Materials and methods

### Human tissue specimens and cell lines

Fresh tissues, consisting of 45 samples of human gastric cancer (22 patients with metastasis and 23 patients without metastasis) and 31 samples of adjacent normal mucosal tissues, were collected from 76 patients who underwent surgery at the Second Affiliated Hospital of Chongqing Medical University (Chongqing, China) between 2012 and 2013. The present study complies with the regulations of the Ministry of Health, the ‘biomedical research involving human ethics review (tentative)’ and the Declaration of Helsinki on the Ethical Principles for Medical Research Involving Human Subjects. All patients provided informed consent according to the protocols approved by the Institutional Review Board of the Second Affiliated Hospital of Chongqing Medical University.

AGS and N87 cell lines were obtained from the American Type Culture Collection (Manassas, VA, USA). MGC80-3, SGC-7901, HGC-27, GES-1 and MKN-45 cell lines were purchased from the Type Culture Collection of the Chinese Academy of Sciences (Shanghai, China). The cell lines were cultured in RPMI 1640 medium (Hyclone, Thermo Scientific, Waltham, MA, USA) supplemented with 10% fetal bovine serum (FBS), and were maintained at 37°C and 5% CO_2_ in an incubator.

### Primers, RNA isolation and miRNA detection

miR-503 and U6 primers were purchased from Takara Bio (Otsu, Japan). Total miRNA was extracted from cultured cells and human tissue specimens with RNAiso for Small RNA (Takara Bio), according to the manufacturer’s instructions. PolyA tails were added to miR-503 and U6 with the miRNA Reaction Buffer Mix (Takara Bio) and cDNA was synthesised from 5 ng total RNA using the miRNA PrimeScriptRT Enzyme Mix (Takara Bio). Quantitative polymerase chain reaction (qPCR) was run on a CFX96™ Real-Time PCR Detection System (Bio-Rad, Hercules, CA, USA) with SYBR^®^ Premix Ex Taq™ II (Takara Bio). The qPCR conditions were as follows: 95°C for 30 sec, followed by 40 cycles of 95°C for 5 sec, then 60°C for 30 sec. The data were normalised against the U6snRNA. Subsequent to amplification, a melting curve analysis was performed to ensure the specificity of the products.

### Oligonucleotide transfection

miR-503 and cont-miR (control-miR) mimics were synthesised by Sangon Biotechnology, Co., Ltd. (Shanghai, China), and mimic cotransfections were performed with Lipofectamine 2000 (Invitrogen Life Technologies, Carlsbad, CA, USA). At 24-h post-transfection, the cells were plated for proliferation, migration and invasion assays. The cells were harvested for RNA and protein analyses at 48 h post-transfection.

### Cell viability and clonability assays

The transfected cells were seeded into 96-well plates at a density of 1×10^4^ cells/well. MTT solution (20 μl of 5 mg/ml) was added to the cultures (200-μl volumes) prior to a 4-h incubation at 37°C. Following removal of the culture medium, the remaining crystals were dissolved in dimethylsulfoxide, and the absorbance at 570 nm was measured. For colony formation assays, the cells were seeded at a low density (1,000 cells/plate) and allowed to grow until visible colonies appeared. The cells were then stained with Giemsa, and the colonies were counted.

### Migration and invasion assays

Cytoselect 24-well cell migration and invasion assay kits (Cell Biolabs, Inc., San Diego, CA, USA) were used for the migration and invasion assays, according to the manufacturer’s instructions. AGS and MKN45 cell lines transfected with miR-503 mimics or control miR were harvested 72 h after transfection and resuspended in serum-free Opti-minimum essential medium. The cells (10×10^4^ per 500 μl serum-free media) were added to the upper chambers, and the lower chambers were filled with 750 μl media with 10% FBS. The cells were incubated for 16 h at 37°C and 5% CO_2_ in a tissue culture incubator. After 16 h, the non-migrated/non-invading cells were removed from the upper sides of the Transwell membrane filter inserts with cotton-tipped swabs. Migrated/invaded cells on the lower sides of the inserts were stained, and the absorbance was read at 560 nm according to the manufacturer’s instructions.

### Antibodies

The anti-fibronectin antibody was obtained from Santa Cruz Biotechnology, Inc.,(Santa Cruz, CA, USA). Antibodies against vimentin, E-cadherin and N-cadherin were purchased from Abcam (Cambridge, UK). Antibodies against SNAIL and GAPDH were purchased from BD Biosciences (Franklin Lakes, NJ, USA). Horseradish peroxidase (HRP)-conjugated goat anti-mouse and anti-rabbit immunoglobulin G (IgG) were purchased from Santa Cruz Biotechnology, Inc.

### Immunoblotting

Total protein was extracted from the transfected cells with radio-immunoprecipitation assay lysis buffer (Beyotime Institute of Biotechnology, Shanghai, China), according to the manufacturer’s instructions. After the whole-cell protein extracts were quantified with a bicinchoninic acid protein assay, equivalent amounts of cell lysates were resolved using 10% SDS-polyacrylamide gel electrophoresis and transferred onto a polyvinylidene fluoride membrane, which was then blocked in 5% skimmed milk in Tris-buffered saline Tween 20 for 1 h at 4°C. The blots were then incubated with the primary antibodies: mouse monoclonal antibody to fibronectin, mouse monoclonal antibody to E-cadherin, rabbit polyclonal antibody to N-cadherin, rabbit polyclonal antibody to SNAIL, mouse monoclonal antibody to GAPDH. Following subsequent incubations with HRP-conjugated goat anti-mouse and anti-rabbit immunoglobulin G (IgG) secondary antibodies, the protein bands were visualised with an enhanced chemiluminescence reagent (Millipore, Billerica, MA, USA). The following antibody dilutions were used: Anti-fibronectin, 1:200; anti-vimentin, anti-E-cadherin and anti-N-cadherin, 1:1200; anti-SNAIL and anti-GAPDH, 1:500; and HRP-conjugated IgG, 1:7000.

### Statistical analysis

SPSS 13.0 software was used for the statistical analysis (SPSS, Inc., Chicago, IL, USA). Data are presented as the mean ± standard deviation. Group comparisons were performed with Student’s t-test, and P<0.05 was considered to indicate a significant difference.

## Results

### miR-503 expression is downregulated in gastric cancer cell lines

**Figure 1:**
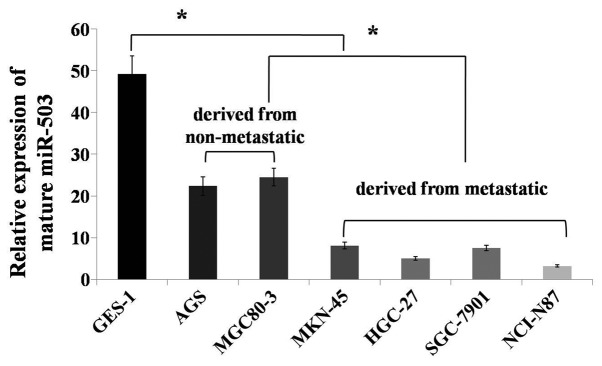
Reduced miR-503 expression in gastric cancer cell lines (SGC7901, HGC27, AGS, MKN45, MGC80-3 and NCI-N87) compared with a normal gastric mucosa cell line (GES1). miR-503 expression was upregulated in the gastric cancer cell lines derived from non-metastatic tissues compared with those derived from metastatic tissues. Data are shown as the mean ± SD (n=6) in the cell lines; ^*^P<0.01. The mature miR-503 expression levels are normalised to U6 small nuclear RNA. SD, standard deviation.

To investigate the miR-503 expression levels in human gastric cancer cell lines, expression was monitored in several cancer cell lines (SGC7901, HGC27, AGS, MKN45, NCI-N87 and MGC80-3) and a normal gastric mucosa cell line (GES1). miR-503 expression was reduced in the gastric cancer cell lines compared with the normal gastric mucosa cells. The AGS and MGC80-3 cell lines were derived from non-metastatic tissues, and the SGC7901, HGC27, MKN45 and NCI-N87 cell lines were derived from metastatic tissues. miR-503 expression was upregulated in the gastric cancer cell lines that were derived from non-metastatic tissues compared with those derived from metastatic tissues (Fig. 1). Thus, miR-503 may play an important role in gastric cancer. To further understand the role of miR-503, its expression was detected in gastric cancer tissues.

### miR-503 expression is downregulated in gastric cancer tissues, and decreased miR-503 expression is associated with gastric cancer metastasis

**Figure 2:**
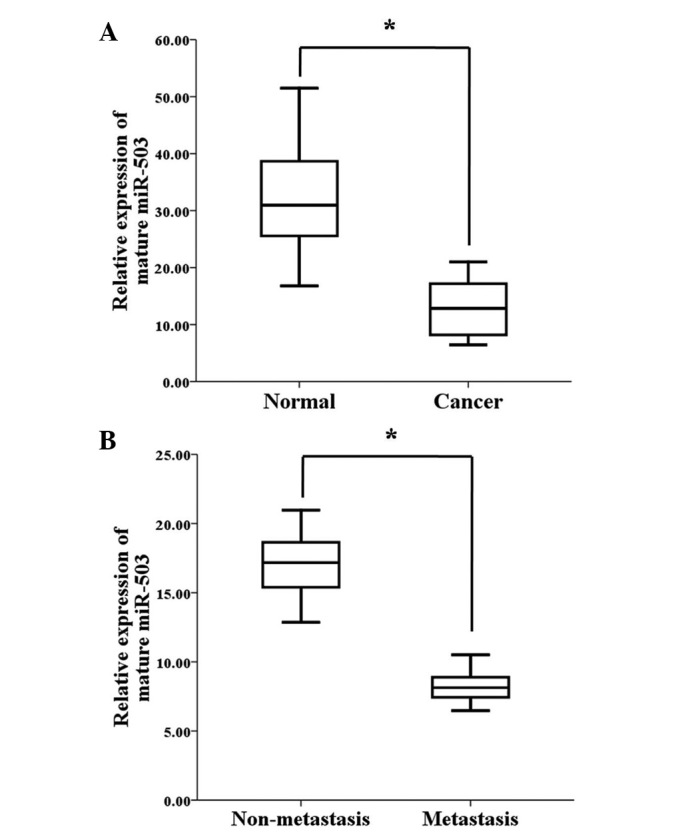
miR-503 expression is downregulated in gastric cancer tissues, and decreased miR-503 expression is associated with gastric cancer metastasis. (A) Mature miR-503 expression levels in fresh gastric cancer (n=45) or fresh adjacent normal mucosa tissues (n=31) were determined by quantitative polymerase chain reaction (qPCR). The mature miR-503 expression levels were significantly lower in gastric cancer tissues compared with the adjacent normal mucosa tissues. (B) Mature miR-503 expression levels in metastatic and non-metastatic gastric cancers. The miR-503 expression levels were significantly lower in patients with metastases compared with patients without metastases. Data are shown separately in human samples; ^*^P<0.01. Mature miR-503 expression levels are normalised to the U6 small nuclear RNA levels.

To explore the role of miR-503 in human gastric cancer development, its expression levels were detected in 45 human gastric cancer tissue samples and 31 adjacent normal mucosa tissue samples. According to the qPCR analysis, the miR-503 expression levels were significantly reduced in the tumour tissues compared with the adjacent normal mucosa tissues (Fig. 2A). To determine whether miR-503 expression is associated with gastric cancer metastasis, the miR-503 expression levels were examined in 45 archived primary gastric tumours. These tumours were divided into two groups; the tumours in one group were resected from 22 patients with lymph node or distant organ metastases, and the tumours in the other group were resected from 23 patients without metastases. According to the qPCR analysis, the miR-503 expression levels were significantly lower in the patients with metastasis when compared with the patients without metastasis (Fig. 2B). These results demonstrate that miR-503 may be a critical miRNA in gastric cancer progression, and that decreased miR-503 expression is associated with gastric cancer metastasis.

### miR-503 inhibits gastric cancer cell migration, invasion and proliferation

**Figure 3:**
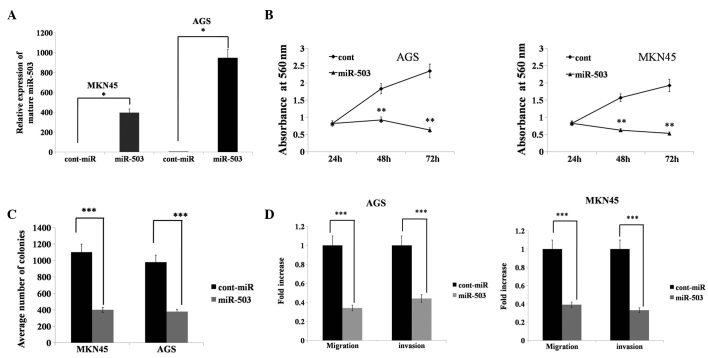
miR-503 inhibits gastric cancer cell migration, invasion and proliferation. (A) Transient transfection of miR-503 mimics significantly increased miR-503 expression in the gastric cancer cells. (B) AGS and MKN45 cell proliferation was significantly reduced after miR-503 transfection compared with cont-miR transfection. (C) miR-503 overexpression significantly inhibited the colony-forming abilities of the gastric cancer cells. (D) miR-503 significantly reduced the migration and invasion capacities of the AGS and MKN45 cells compared with the controls. ^*^P<0.001, ^**^P<0.05 and ^***^P<0.01. cont, control.

To determine the functional significance of miR-503 overexpression in gastric cancer, the AGS and MKN45 gastric cancer cell lines were transfected with miR-503 mimics. miR-503 was significantly overexpressed in the AGS and MKN45 cells lines following miR-503 mimic transfection compared with the cont-miR-transfected cells (Fig. 3A). Forced miR-503 expression significantly decreased cell proliferation relative to cont-miR expression (Fig. 3B). miR-503-transfected cells also exhibited a reduced colony-forming ability, as the number of foci in the miR-503-expressing cells was reduced when compared with the cont-miR-transfected cells (Fig. 3C). Transwell migration and Matrigel invasion assays demonstrated that miR-503 significantly reduced the migration and invasion capacities of the AGS and MKN45 cells (Fig. 3D). These results demonstrated that miR-503 acts as a tumour suppressor gene in gastric cancer.

### miR-503 promotes an epithelial phenotype in gastric cancer

**Figure 4:**
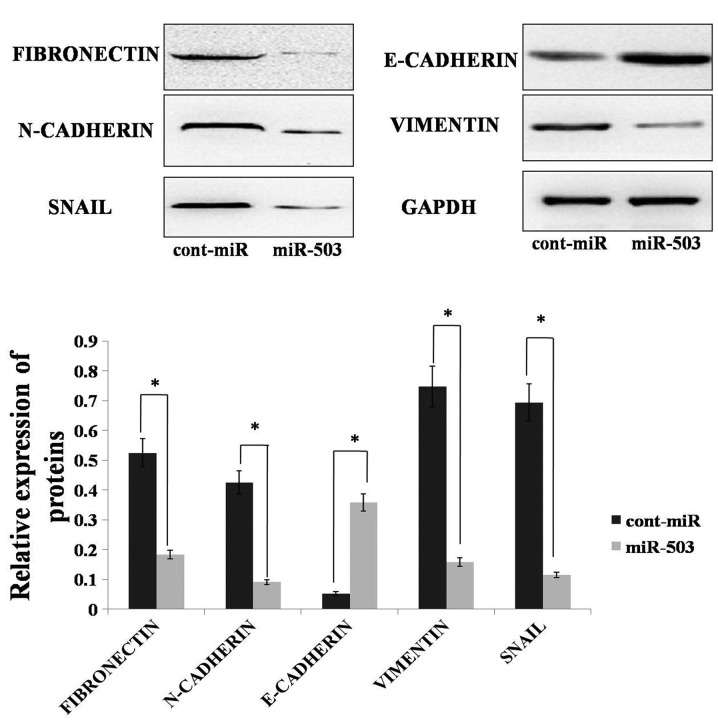
miR-503 promotes an epithelial phenotype in gastric cancer. Mesenchymal markers were analysed in the AGS cell line by immunoblotting following transfection with miR-503 mimics or cont-miR. Data are shown as the mean ± SD; n=6. ^*^P<0.01 compared with controls. cont, control; SD, standard deviation.

As miR-503 can inhibit gastric cancer cell migration and invasion, we hypothesised that miR-503 could inhibit EMT in gastric cancer. To determine if molecular changes typical of reduced EMT occurred in miR-503-expressing cells, the expression of mesenchymal markers, including fibronectin, vimentin, N-cadherin, SNAIL and the epithelial marker, E-cadherin, was examined in the AGS cell line. Immunoblot analysis showed that the expression levels of fibronectin, vimentin and SNAIL were decreased in the AGS cell line with forced miR-503 expression. The N-cadherin protein level was also decreased in the AGS cell line with forced miR-503 expression. Furthermore, forced miR-503 expression increased E-cadherin expression in the AGS cells, whereas the control transfected cells remained E-cadherin-negative (Fig. 4). Altogether, these results indicated that miR-503 could inhibit EMT in gastric cancer cells.

## Discussion

miR-503 is expressed differently in various types of cancer; in certain types, miR-503 expression is upregulated. The miR microarray technique has been used to demonstrate that miR-503 is upregulated in human retinoblastoma tissues (20). In addition, miR-503 expression has been shown to be upregulated in human parathyroid carcinomas (21). Additionally, high miR-503 expression has been significantly associated with shorter overall survival in patients with adrenocortical carcinoma (22). However, all of these results were obtained with the miR microarray technique and were not confirmed by qPCR. A concrete mechanism to explain why miR-503 is upregulated in certain cancers and the identity of the target gene of miR-503 remains unknown.

In other types of cancer, miR-503 expression is downregulated. In oral cancer, miR-503 has been found to be downregulated, according to the miR microarray technique (23). In addition, miR-503 has been detected in non-metastatic prostate cancer xenografts, but not in metastatic grafts (24). Jiang *et al* identified the target gene of miR-503 and delineated its function. The study revealed that miR-503 could silence cyclin D1 (CCND1), a well-known proto-oncogene that is implicated in a variety of cancer types. Thus, miR-503 could reduce S-phase cell populations and cause cell growth inhibition (25). Xu *et al* also identified that miR-503 directly targeted CCND1, and that abnormal miR-503 suppression led to elevated CCND1 levels, which may promote EEC carcinogenesis and progression (17). Furthermore, miR-503 was identified as a cell cycle regulator with involvement in cell adhesion, migration and angiogenesis processes (26). This finding indicates that miR-503 may be a putative tumour suppressor. Zhou *et al* identified the reason behind the downregulation of miR-503 expression in human hepatocellular carcinomas (HCC) as namely due to the epigenetic modulation of its promoter (27) The results fully validated the finding that miR-503 was downregulated in certain types of cancers and elucidated the reason for the downregulation of miR-503 and the identity of the miR-503 target gene.

In the present study, miR-503 expression was found to be reduced in gastric cancer cell lines compared with normal gastric mucosa cell lines. Additionally, the miR-503 expression levels were significantly reduced in tumour tissues compared with the adjacent normal mucosa tissues. Meanwhile, the miR-503 expression levels in metastatic patients were significantly lower than those in non-metastatic patients. The results showed that miR-503 expression was correlated with gastric carcinoma progression. Several studies have agreed with these results. A study by Zhou and Wang identified that miR-503 was significantly downregulated in HCCLM3, an HCC cell line with a strong metastatic potential, when compared with MHCC97-L, an HCC cell line with a lower metastatic potential. Decreased miR-503 expression was identified in HCC cell lines with high metastatic abilities. Meanwhile, overexpressed miR-503 inhibited the proliferation and metastasis of HCCLM3 cells *in vitro* (28). In the present study, forced miR-503 expression was found to significantly decrease cell migration, invasion and proliferation relative to cont-miR expression. The reason why forced miR-503 expression inhibits gastric cancer cell migration, invasion and proliferation is not fully understood. This finding could be the result of several factors. First, one target of miR-503 is cyclin D1, which can promote cancer cell growth. Thus, when miR-503 is overexpressed in cancer cells, cyclin D1 expression is downregulated and cell growth is subsequently inhibited. Second, increased miR-503 expression impairs endothelial cell networking capacities, indicating an anti-angiogenic role for this particular miR (26). Zhou *et al* also identified that miR-503 directly targets and negatively regulates the expression of vascular endothelial growth factor (VEGF)-A and fibroblast growth factor (FGF)2. As VEGF-A and FGF2 are important angiogenic factors, miR-503 can suppress tumour growth by inhibiting angiogenesis (27).

In gastric cancers, EMT is known to be associated with a migratory phenotype. Kurashige *et al* identified that miR-200b regulates cell proliferation, invasion and migration by directly targeting ZEB2 in gastric carcinomas (29). Zhang *et al* revealed that miR-27 promotes human gastric cancer cell metastasis by inducing EMT (30). However, it remains unknown whether miR-503 can inhibit EMT in human gastric cancers. We predicted that Notch and IGF1R, which are EMT regulators, may be target genes of miR-503. In the present study, it was identified that miR-503 overexpression could inhibit EMT in gastric cancer cells. Therefore, these results show that miR-503 may act as an important regulator of EMT in gastric cancer. In a forthcoming study, we will determine whether Notch and IGF1R are target genes of miR-503 with luciferase reporter assays, and the miR-503 regulatory mechanism will be studied further by xenograft experiments.

In conclusion, the results of the present study have identified miR-503 as a novel tumour suppressor gene in gastric cancer. Furthermore, the data have demonstrated that miR-503 can curb gastric cancer cell metastasis by inhibiting EMT. These results indicate potential applications for miR-503 in gastric cancer therapy.
